# Pandora's Box

**DOI:** 10.1192/bji.2024.40

**Published:** 2025-02

## Fat cells do not forget!

You have finally reached your target weight after weeks of coping with hunger on a strict diet. You can finally relax and savour some of your favourite dishes again. But your joy is short-lived, as in no time the lost weight piles up once more. Stop beating yourself up about it; you are indeed lacking enough self-discipline, but your body has a lot to answer for, and the main culprit is your fat cells and their long memory!

On a more serious note, obesity is a major health hazard associated with diabetes and cardiovascular problems and many other ill-health states. Various drugs are available, and many people manage to lose weight, only to start piling it up as soon as they relax their diet. The body's epigenetic obesogenic memory, a defence mechanism against weight changes, has attracted much research interest in recent years, and unravelling this process could help deal with the difficulty of maintaining weight loss. Whereas previous work to understand this process used bulk tissues or whole blood, a recent study published in *Nature* focused on adipose tissue from humans and mice.

The investigators obtained biopsied subcutaneous and omental fat tissue from humans, including individuals who had never been obese and those who had lived with obesity, before and 2 years after significant weight loss, as well as fat tissue from lean, obese and formerly obese mice. Using single-nucleus RNA sequencing, they confirmed the presence of post-weight-loss retention of transcriptional cellular changes in both humans and mice. Most importantly, they found changes induced by obesity in the epigenomes of the mouse fat cells that persisted and impaired the function of the cells and their response to metabolic stimuli. The mice that carried this obesogenic memory showed accelerated rebound weight gain. Targeting these obesogenic memory mechanisms alongside healthy eating and general lifestyle after weight loss may be key to maintaining a healthy weight and achieving better long-term health.

Hinte LC, Castellano-Castillo D, Ghosh A, Melrose K, Gasser E, Noé F, et al Adipose tissue retains an epigenetic memory of obesity after weight loss. Nature 2024; 636: 457–65.39558077 10.1038/s41586-024-08165-7PMC11634781

## Of men and women in pain

We know that the opioid system is involved in pain control in humans, and powerful prescribed analgesics target this system. Previous studies have shown that meditation can be beneficial in relieving at least some forms of pain. Women are more likely to suffer with chronic pain, and they are less responsive to opioid analgesics. You may be interested to know that these gender differences are also present in rodents, in which it has been shown that blockade of endogenous opioidergic systems reverses analgesia in males but not in females. A study published in *PNAS Nexus* examined the possibility that such dimorphic, sex-specific differences may also apply to humans.

Male and female study participants were randomised to mindfulness meditation or sham mindfulness meditation. After training in this, they were exposed to pain (via a noxious but safe heat stimulus) and asked to practise their allocated meditation, while receiving either naloxone or saline intravenously. In both men and women who received the saline infusion, mindfulness meditation lowered the intensity of the noxious-heat-induced pain. Intravenous naloxone, which blocks the opioid effect, reversed meditation-induced analgesia in men but failed to do so in women. These results suggest that unlike in men, self-regulated analgesia in women is not mediated by the endogenous opioid system. This highlights the need to explore other systems potentially involved in regulating pain in women. Better understanding of mechanisms relevant to women could lead to much-needed more effective analgesics.

Dean JG, Reyes M, Oliva V, Khatib L, Riegner G, Gonzalez N, et al Self-regulated analgesia in males but not females is mediated by endogenous opioids. PNAS Nexus 2024; 3(10): e453.10.1093/pnasnexus/pgae453PMC1148987139430222

## The pregnant female brain

A pregnant woman's body undergoes significant physiological changes, including massive increases in production of hormones – in particular, oestrogen and progesterone – known to have neuromodulatory properties. Steroid hormones drive neurogenesis and other neuronal, microglial and astrocyte cellular changes associated with neuroplasticity. A reduction in cortical thickness has been reported in the third trimester of pregnancy. It is important to seek a better understanding of pregnancy-related brain changes to enable better understanding of the possible pathophysiological mechanisms involved; this will hopefully lead to better and earlier detection of perinatal mental health conditions.

Researchers from the University of California and the National Institute of Mental Health, Bethesda, USA, endeavoured to map neuroanatomical changes in the brain from preconception to childbirth. They recruited a healthy 38-year-old primiparous woman, who volunteered to undergo a series of 26 magnetic resonance imaging (MRI) brain scans, starting 3 weeks preconception and continuing to 2 years postpartum. Alongside this, they collected blood samples for hormone estimation.

The MRI images showed widespread decreases in cortical thickness and grey matter volume, which become more prominent as the pregnancy progressed. There were also reductions in grey matter volume within the hippocampus and parahippocampal cortex, as well as other areas including the thalamus, hypothalamus, substantia nigra, caudate and brain stem. By contrast, there were nonlinear increases in the white matter of the brain. As the pregnancy progressed, a dramatic rise in levels of sex hormones was observed alongside the brain changes, and these levels dropped precipitously after childbirth. The researchers compared the imaging changes with those of eight healthy controls to preclude the possibility of their being within the range of normal brain variability. The grey and white matter changes in the brain of the pregnant participant were 3–4 times greater than those of the controls.

These findings, alongside existing evidence from non-human mammals, demonstrate the brain's remarkable capacity for neuroplasticity. Notably, some of these changes, including the reductions in grey matter volume and cortical thickness, persisted at 2 years after childbirth, whereas the white matter changes did not. This precision imaging study, as the researchers called it, showed gradual and progressive changes in grey and white matter in step with the progression of the pregnancy. They considered the possibility that an increase in cerebrospinal fluid volume during the pregnancy due to water retention may have caused compression of cortical tissue, but the persistence of the brain changes 2 years postpartum did not support this.

A lot more research is needed to understand the relationship between brain changes and brain fluid volume and hormonal changes and their functional significance in relation to parental behaviour and vulnerability to mental illness. Investigation of concomitant cellular changes in neuronal, glial and astrocyte systems, alongside imaging, would help to unravel the mechanisms involved and their relevance to normal and pathological emotional, behavioural and other neuropsychiatric states during pregnancy and the perinatal period.

Pritschet L, Taylor CM, Cossio D, Faskowitz J, Santander T, Handwerker DA, et al Neuroanatomical changes observed over the course of a human pregnancy. Nat Neurosci 2024; 27: 2253–60.39284962 10.1038/s41593-024-01741-0PMC11537970

## The pregnant male brain

To be fair to men, it should be noted that they also go through a pregnancy by proxy, and I don't mean the Couvade syndrome. There is research into changes in the paternal brain after childbirth showing increased activity in the caregiving brain network.

Bottemanne and Joly set out to examine any possible changes in expectant fathers’ brains during their partners’ pregnancies. They reviewed studies that showed a number of interesting changes. Decreases in testosterone were reported, which were correlated with brain responses to the infant after childbirth, as well as correlations between gestational age and activation in the left inferior frontal gyrus and the amygdala. Animal studies were also reviewed; the overall findings were that expectant fathers experience hormonal and neuroplastic changes in their brain in anticipation of the birth of the child. The authors recommend further research into the area, noting that 8% of fathers develop postpartum depression in the year of childbirth.

Bottemanne H, Joly L. How the paternal brain is wired by pregnancy. JAMA Psychiatry [Epub ahead of print] 13 Nov 2024. Available from: 10.1001/jamapsychiatry.2024.3592.39535755

## Hormonal synchrony in expecting parents

Hormonal changes are known to occur in both expecting parents, and the authors of a systematic review addressed the question of whether hormonal synchronisation may occur between them. Using data from 13 eligible studies, they found that expectant parents displayed linkages in cortisol, testosterone and progesterone concentrations in the prenatal period. There was also synchrony of oxytocin, testosterone and cortisol up to 2 years after birth. They interpreted these findings as being of functional significance in adaptation to parental behaviour, promotion of the romantic bond and infant development. Is this synchrony present in non-harmonious parental relationships?

Daneshnia N, Chechko N, Nehls S. Do parental hormone levels synchronize during the prenatal and postpartum periods? A systematic review. Clin Child Fam Psychol Rev 2024; 27(3): 658–76.38615295 10.1007/s10567-024-00474-7PMC11486823

